# Genetic variants associated with physical performance and anthropometry in old age: a genome-wide association study in the *ilSIRENTE* cohort

**DOI:** 10.1038/s41598-017-13475-0

**Published:** 2017-11-20

**Authors:** David Heckerman, Bryan J. Traynor, Anna Picca, Riccardo Calvani, Emanuele Marzetti, Dena Hernandez, Michael Nalls, Sampath Arepali, Luigi Ferrucci, Francesco Landi

**Affiliations:** 1 Microsoft Research, Los Angeles, California USA; 2 Neuromuscular Diseases Research Section, Laboratory of Neurogenetics, National Institute on Aging, 35 Convent Drive, Room 1A-1000, Bethesda, MD 20892 USA; 3 Center for Geriatric Medicine (CEMI), Department of Geriatrics, Neurosciences and Orthopedics, Catholic University of Sacred Heart, Rome, 00168 Italy; 4 Genomics Technology Group, Laboratory of Neurogenetics, National Institute on Aging, 35 Convent Drive, Room 1A-1000, Bethesda, MD 20892 USA; 5 Molecular Genetics Unit, Laboratory of Neurogenetics, National Institute on Aging, 35 Convent Drive, Room 1A-1000, Bethesda, MD 20892 USA; 6 Longitudinal Studies Section, Clinical Research Branch, National Institute on Aging, 251 Bayview Blvd., Room BRC/04C225, Baltimore, MD 21224 USA

## Abstract

Unraveling the complexity of aging is crucial for understanding its mechanisms and its role as a risk factor for most chronic conditions. Advancements marked by genome-wide association studies (GWASs) have sparked interest in gene cataloguing in the context of aging and age-related conditions. Here, we used GWAS to explore whether single nucleotide polymorphisms (SNPs) were associated with functional and anthropometric parameters in a cohort of old community-dwellers enrolled in the *ilSIRENTE* study. Analyses were carried out in men and women aged 80+ years enrolled in the *ilSIRENTE* study (n = 286) and replicated in the *inCHIANTI* study (n = 1055). Genotyping was accomplished on Infinium Human610-QUAD version 1. In the *ilSIRENTE* population, genetic variants in ZNF295 and C2CD2 (rs928874 and rs1788355) on chromosome 21q22.3, were significantly associated with the 4-meter gait speed (rs928874, p = 5.61 × 10^−8^; rs1788355, p = 5.73 × 10^−8^). This association was not replicated in the *inCHIANTI* population. Our findings suggest that specific SNPs may be associated with a key measure of physical performance in older adults. GWASs using larger samples are needed to confirm these preliminary results to enhance our comprehension of complex age-associated phenomena.

## Introduction

Advancements in healthcare, nutrition and sanitation have remarkably extended the average human life expectancy. Thus, the elderly population has expanded considerably in Western countries. One of the most notable consequences of this demographic shift is the increased prevalence of chronic degenerative diseases and functional impairments. In such a scenario, the promotion of strategies aimed at preserving physical function and preventing its deterioration toward disability is crucial for the sustainability of healthcare systems^1,2^. Unfortunately, little is known about the mechanisms by which aging increases susceptibility to chronic diseases and disability, which hampers the identification of suitable therapeutic targets.

It is traditionally assumed that successful aging results from the combination of genetic and environmental factors^3^. Genome-wide association studies (GWASs) have been proposed as a valuable strategy for identifying genetic variants associated with positive or negative health phenotypes, such as longevity and age-related diseases^4–6^. For instance, GWASs allowed for the identification of polymorphisms associated with top five leading causes of death in adulthood and advanced age (i.e., heart disease, cancer, stroke, Alzheimer’s disease, and diabetes)^7–11^.

**Figure 1:**
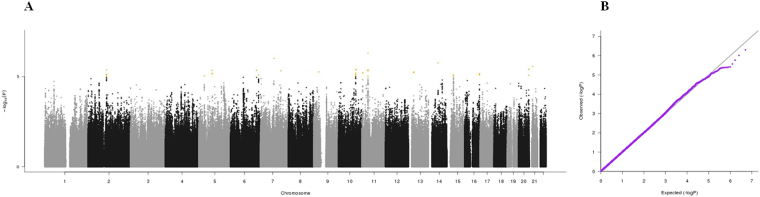
(**A**) Manhattan plot and (**B**) QQ plot of p-values based on analysis of the *ilSIRENTE* activities of daily living (ADL) data.

**Figure 2:**
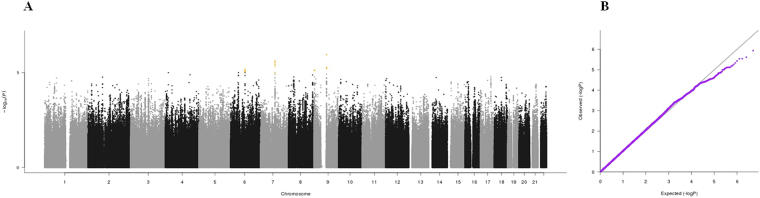
(**A**) Manhattan plot and (**B**) QQ plot of p-values based on analysis of the *ilSIRENTE* calf circumference data.

**Figure 3:**
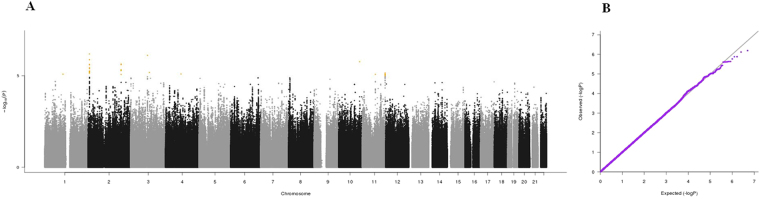
(**A**) Manhattan plot and (**B**) QQ plot of p-values based on analysis of the *ilSIRENTE* mid-arm muscle circumference data.

The importance of unraveling disease-associated gene variants resides in their possible exploitation for treatment purposes. For example, the discovery of the genes responsible for maturity-onset diabetes of the young (MODY, which is considered to be a form of progeria)^12^ has allowed for selecting patients whose glycemia is better controlled on sulphonylureas than insulin^13^.

Several age-related phenotypes (e.g., pulmonary function, fasting glucose, bone mineral density, cognitive function) show moderate to high heritability^14–16^. In addition, genetic background has been proposed to contribute up to approximately 65% of the variability in muscle mass and strength^17,18^. GWASs have subsequently identified specific single-nucleotide polymorphisms (SNPs) associated with muscle mass, strength and physical function in late life (reviewed by^19^). In particular, SNPs pertaining to angiotensin-converting enzyme (ACE) gene I/D, α-actinin-3 (ACTN3) R577X, and myostatin (MSTN) K153R have been extensively studied^19^. With the exception of MSTN K153R, which seems to be associated with a sarcopenic phenotype in very old women^20^, no conclusive findings have been obtained for other candidate genetic variants^19^.

The present study was therefore undertaken to identify specific genetic variants associated with measures of functional status, physical performance, muscle mass, muscle strength, and cognition in a well-characterized and genetically homogeneous cohort of octogenarians and nonagenarians living in a mountain community in Central Italy, enrolled in the *ilSIRENTE* study (“Invecchiamento e Longevità nel Sirente”)^21^. The longitudinal design of the study enabled us to explore whether specific SNPs were associated with changes in the variables of interest over time.

## Results

DNA was purified from 286 participants. Age and gender distribution as well as anthropometric and functional characteristics were unvaried between the subset under investigation and the enrollees from whom DNA was unavailable (data not shown). The main characteristics of the subsample used for the present study are shown in Table 1 along with those of the *inCHIANTI* (“Invecchiare in Chianti”) replication population^22^.

**Table 1:** Main characteristics of the *ilSIRENTE* and *inCHIANTI* populations.

Characteristic	*ilSIRENTE* (n = 286)	*inCHIANTI* (n = 1055)
**Age**, *years*	86.1 ± 4.9	67.8 ± 15.7
**Female**	170 (64)	615 (58)
**Education**, *years*	5.0 ± 1.6	8 ± 1.6
**Number of diseases**	1.9 ± 1.3	1.1 ± 0.8
**Specific diseases**		
Hypertension	206 (72)	484 (46)
Ischemic heart disease	34 (12)	89 (8)
Congestive heart failure	18 (6)	32 (3)
Cancer	14 (5)	57 (5)
Chronic obstructive pulmonary disease	37 (13)	65 (6)
Diabetes	83 (29)	127 (12)
Cerebrovascular diseases	15 (5)	35 (3)
Osteoarthritis	53 (18)	188 (18)
**Number of medications**	3.3 ± 2.3	2.0 ± 1.3
**BMI, kg/m** ^**2**^	25.3 ± 4.4	27.2 ± 4.1
**Physical performance measures**		
Short Physical Performance Battery summary score	6.6 ± 3.9	10.2 ± 3.1
4-m walking speed (m/s)	0.48 ± 0.31	1.04 ± 0.28
Handgrip strength (kg)	28.9 ± 14.6	30.7 ± 14.1
**Functional status measures**		
ADL score	1.5 ± 2.5	0.25 ± 1.0
IADL score	3.1 ± 2.5	0.86 ± 2.1
**CPS** score	0.9 ± 1.5	—
**MMSE** score	—	26.2 ± 2.8
**Hematological parameters**		
Albumin, g/dL	4.2 ± 0.3	4.3 ± 0.3
Cholesterol, mg/dL	197.1 ± 44.1	214.9 ± 40.5
C-reactive protein, µg/mL	4.1 ± 3.4	4.9 ± 9.0
Interleukin 6, pg/mL	3.0 ± 2.6	2.1 ± 3.9
TNF-α, pg/mL	1.8 ± 2.2	5.0 ± 2.5

Data are given as number (percent) for gender and specific diseases; all other variables are reported as mean ± standard deviation.

*Abbreviations*: ADL: activities of daily living; BMI: Body Mass Index; CPS: Cognitive Performance Scale; IADL: instrumental activities of daily living; MMSE: MiniMental State Examination; TNF: tumor necrosis factor.

**Figure 4:**
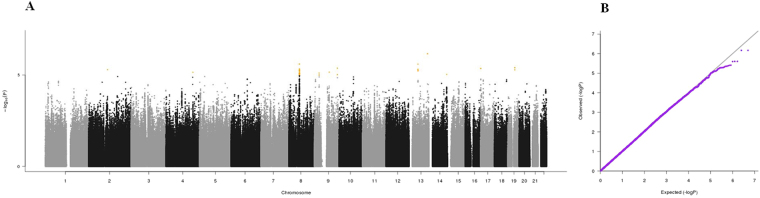
(**A**) Manhattan plot and (**B**) QQ plot of p-values based on analysis of the *ilSIRENTE* handgrip strength data.

**Figure 5:**
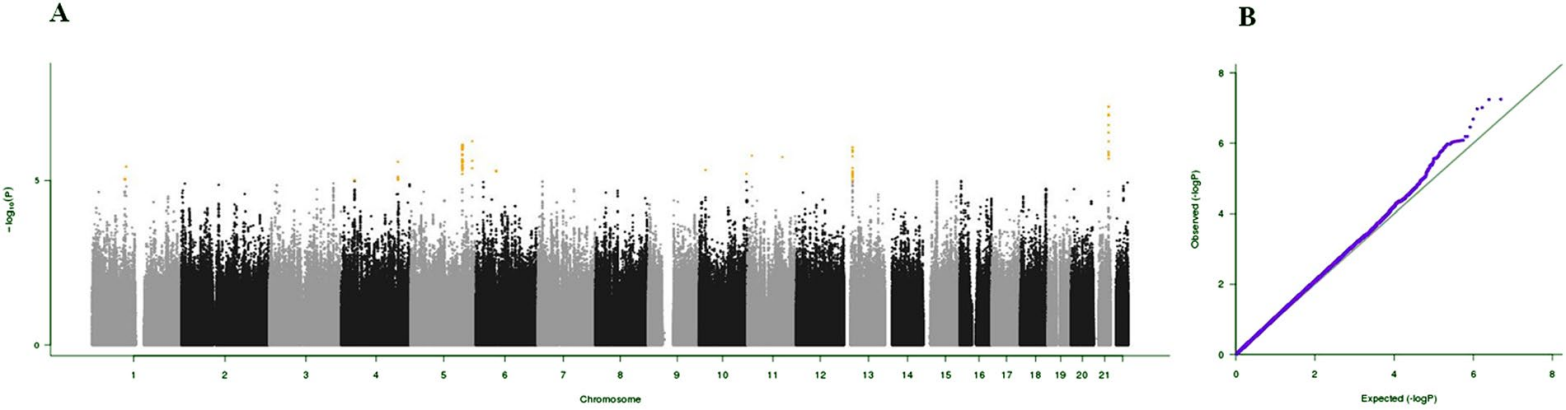
(**A**) Manhattan plot and (**B**) QQ plot of p-values based on analysis of the *ilSIRENTE* 4-meter walk test data.

**Figure 6:**
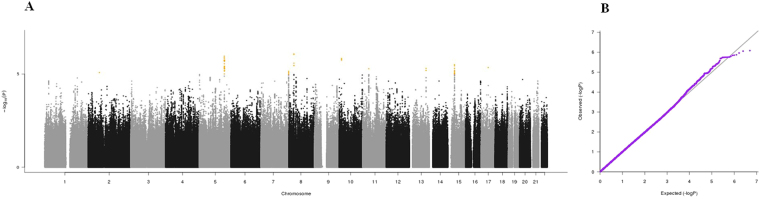
(**A**) Manhattan plot and (**B**) QQ plot of p-values based on analysis of the *ilSIRENTE* Short Physical Performance Battery (SPPB) summary score data.

**Figure 7:**
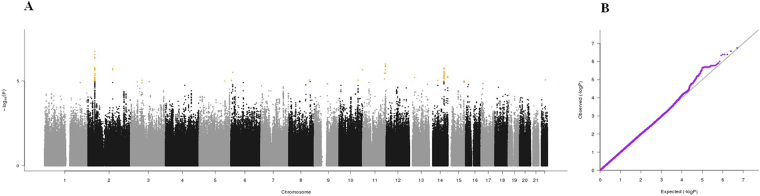
(**A**) Manhattan plot and (**B**) QQ plot of p-values based on analysis of the *ilSIRENTE* 4-meter walk test data over two years.

**Figure 8:**
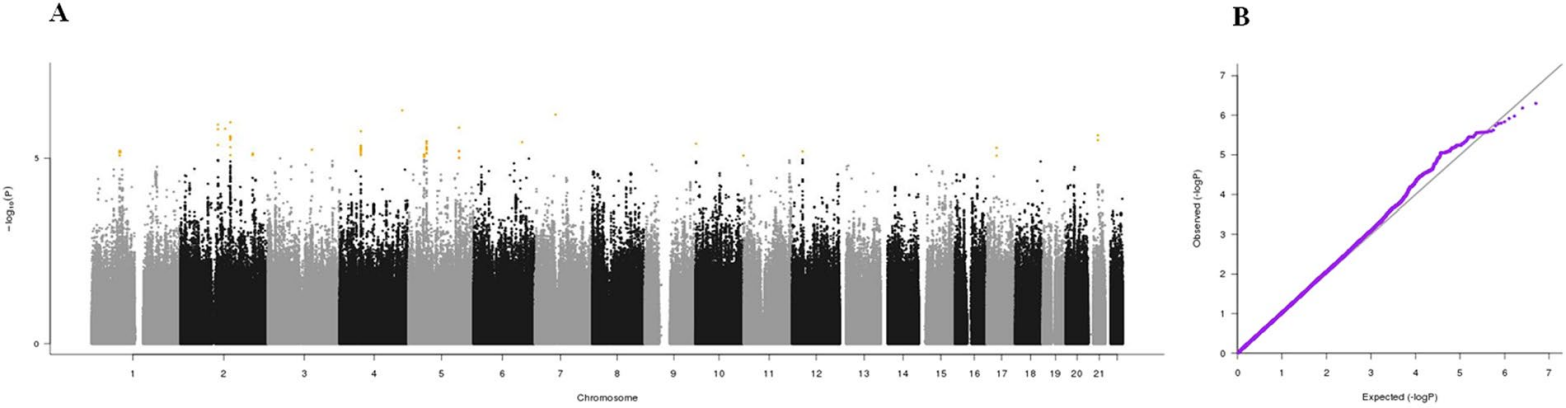
(**A**) Manhattan plot and (**B**) QQ plot of p-values based on analysis of the *ilSIRENTE* Short Physical Performance Battery (SPPB) summary score data over two years.

**Figure 9:**
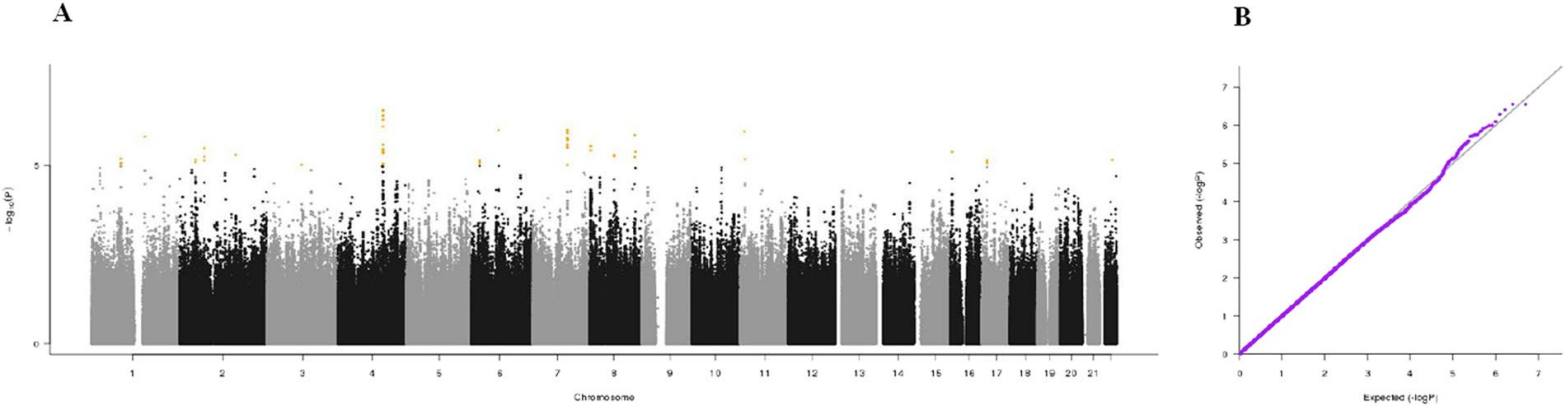
(**A**) Manhattan plot and (**B**) QQ plot of p-values based on analysis of the *ilSIRENTE* Cognitive Performance Scale (CPS) summary score data over two years.

Manhattan plots, QQ plots and top hits list [summary statistics for the single-nucleotide polymorphisms (SNPs) showing p-values < 10^−5^] were generated for the analyses of association between genetic data and activities of daily living (ADL) score^23^, calf circumference (CC)^24^, mid-arm muscle circumference (MAMC)^24^, handgrip strength, 4-meter walk test, Short Physical Performance Battery (SPPB) summary score^25^, Cognitive Performance Scale (CPS) score^26^, as well as for changes in SPPB and 4-meter walk test over two years (Figs 1–9).

In the Manhattan plots, genome-wide significant SNPs are color-coded in red (p values < 5 × 10^−8^), whilst those displayed in orange showed p values < 10^−5^ >5 × 10^−8^. The genomic inflation factor (lambda) for each GWAS analysis, ranging from 0.985 to 1.072, is shown in the QQ plots (Figs1–9). We identified a single association peak that exceeded the Bonferroni’s threshold for significance (Fig.7). This peak was detected in chromosome 21q22.3 in the analysis of 4-meter gait speed (rs928874, p = 5.61 × 10^−8^; rs1788355, p value = 5.73 × 10^−8^), located within a non-coding exon at the start of the *ZNF295* gene and the intergenic region upstream the same gene. The association signal was located in a 95-kb block of linkage disequilibrium that contains two genes: *ZNF295* and *C2CD2*. To further test the association of this region with the 4-meter walk speed, we analyzed rs928874 in an independent replication dataset obtained from the *inCHIANTI* cohort. Rs928874 failed to reach significance in participants either over 80 years (n = 184, p = 0.51) or over 65 years (n = 871, p = 0.294).

## Discussion

In high-income countries, the proportion of people aged over 60 years is growing faster than any other age group, as a result of both longer life expectancy and declining fertility rates. Although population aging can be seen as a success story for public health policies and socioeconomic development, it also implies that chronic age-related diseases and functional impairment have emerged as a major challenge in the 21st century^27^. In such a scenario, a deeper understanding of the biology of aging may provide fundamental insights into the pathogenesis of age-associated conditions, such as declining physical performance, dementia, diabetes, cardiovascular disease and cancer^28^.

The determinants of aging and longevity are not completely known; yet, genetics are thought to play a significant role in successful aging^29^. The development of efficient, affordable, high-density SNP genotyping technologies has provided the opportunity to undertake GWASs to screen large numbers of samples for genetic variations associated with positive or negative health phenotypes^30^. In particular, the possibility of identifying genetic variants associated with skeletal muscle phenotypes in older people has inspired several studies as the decline in muscle mass and strength (i.e., sarcopenia) is a major contributing factor to negative outcomes in late life.

In the present work, we present the results of a GWAS of aging phenotypes in a cohort of old and very old community-living persons enrolled in the *ilSIRENTE* study. This cohort is ideal for research on aging because the population has been thoroughly characterized through the collection of comprehensive information over a long follow-up^21^. Furthermore, the genetically homogeneous nature of this region facilitates the identification of possible genetic variants. For the present investigation, analyses were designed to identify SNPs associated with quantitative aging phenotypes, focused on functional status, physical performance, anthropometry, and cognition (i.e., ADL score, CC, MAMC, handgrip strength, SPPB summary score, 4-meter walk test, and CPS score). We also sought to determine if specific genetic variants were associated with changes in the SPPB summary score and 4-meter gait speed over two years. A single association peak in chromosome 21q22.3 was found with 4-meter gait speed test (rs928874, p = 5.61 × 10^−8^; rs1788355, p = 5.73 × 10^−8^; Fig. 7). Within this locus falls the ZNF295 gene that encodes a zinc finger protein implicated in transcriptional regulation. This same locus was previously linked with handgrip strength in middle-aged and older adults enrolled in the Hunter Community Study^31^. Remarkably, the 21q22.3 region belongs to a group of newly-identified loci associated with age-related diseases, including diabetes mellitus, neurodegeneration, cancer, and cardiovascular disease^32^. SNPs in these loci fall within regulatory regions that modulate the expression of nearby genes^32^.

The association between rs928874 found in our cohort and 4-meter walk speed was not confirmed in the *InCHIANTI* population either in participants over 80 years (n = 184, p = 0.51) or 65–80-year-old enrollees (n = 871, p = 0.294). One possible explanation for such a discrepancy may reside in differences in the linkage disequilibrium levels of rs928874 between the two cohorts^33^. Differences in participant testing procedures and in the covariates used for adjusting the analyses might provide an additional explanation for the lack of concordance between the two populations. On the other hand, we cannot rule out the possibility that the sample size was insufficient for capturing low-frequency variants as well as polymorphisms associated with subtle effects. In such a context, meta-analytical approaches may be able to provide a more comprehensive screening of low-frequency genetic variants associated with aging and capture their pleiotropic effects^32^. Yet, a recent meta-analysis of gait speed GWASs conducted in 31,478 older adults from 17 cohorts of the CHARGE consortium did not find any genome-wide significant signals^34^.

It should be considered that complex age-related phenotypes, such as those investigated in the present work, are influenced by a wealth of biological pathways involving multiple genes. The individual effects of SNPs may therefore be expected to be very small and difficult to identify. To further complicate the matter, the impact of genetic factors is likely modulated by social and environmental factors or may be mediated by some other endophenotypes, including inflammation^32,35^. As such, the association of genetic variants with certain phenotypes may be environment-specific and therefore hardly replicable across cohorts^36,37^. In this respect, it is worth noting that education, the prevalence of certain disease conditions, circulating levels of tumor necrosis factor-alpha (TNF-α), and physical performance were dissimilar between the two cohorts under study (Table 1).

In order to accurately evaluate the staging of biological age and related clinical conditions, as well as the rates of decline of strength and function, the participants from *ilSIRENTE* study underwent evaluations at several time points. As a result, the generation of longitudinal trajectories of biological variables together with age-related outcomes (cardiovascular diseases, dementia, fractures, etc.), and genetic variants associated with aging may help identify factors amenable to interventions aimed at improving the quality of life of older adults. In particular, the existence of SNPs linked with physical performance suggests the intriguing possibility of identifying individuals at risk of functional impairment at an early stage, therefore allowing for interventions to be implemented in a timely manner. The accomplishment of this challenging task requires ad hoc designed studies to conclusively establish if specific genetic variants are indeed associated with relevant aging phenotypes and identify the biological networks governing such complex traits.

## Methods

### Study population

The *ilSIRENTE* study is a prospective cohort study conducted in the mountain community living in the Sirente geographic area (L’Aquila, Abruzzo) in Central Italy (GPS coordinates: 42.349918, 13.399544). The study was designed by the Department of Geriatrics, Neurosciences and Orthopedics of the Catholic University of the Sacred Heart (Rome, Italy) and developed by the teaching nursing home “Opera Santa Maria della Pace” (Fontecchio, L’Aquila, Italy), in a partnership with local administrators and primary care physicians of Sirente Mountain Community Municipalities.

The *ilSIRENTE* study protocol is detailed elsewhere ^21^. Briefly, a preliminary list of persons living in the Sirente area was obtained at the end of October 2003 from the Registry Offices of the 13 municipalities involved in the study. Potential participants were identified by selecting all persons born in the Sirente area before January 1^st^ 1924 and living locally at the time of the survey. Among the eligible persons (n = 429), the refusal rate was very low (16%). Age and gender distribution were not different between people who refused to participate and the enrollees. The overall sample population enrolled in the *ilSIRENTE* study consisted of 364 persons. For the study we considered 286 participants for whom blood sample for DNA extraction was available. The study protocol was approved by the Catholic University’s Ethics Committee. All methods were performed in accordance with the relevant guidelines and regulations and an informed consent was obtained from all participants or from their relatives in the case of cognitive impairment^ 21^.

### Clinical assessment, anthropometry and functional testing

The Minimum Data Set for Home Care (MDS-HC) form was administered to all participants ^26^ to collect data on 350 parameters including socio-economic variables, physical and cognitive status, and major clinical diagnoses.

Muscle mass was estimated from CC and MAMC using a non-elastic flexible plastic tape, as detailed previously ^24^. CC was measured on the left leg (or the right leg for left-handed persons) in a sitting position with the knee and ankle at a right angle and feet resting on the floor. CC was taken at the point of greatest circumference, without compressing subcutaneous tissues. MAMC was calculated using the standard formula^ 38^:

$${\rm{M}}{\rm{A}}{\rm{M}}{\rm{C}}={\rm{m}}{\rm{i}}{\rm{d}}-{\rm{a}}{\rm{r}}{\rm{m}}\,{\rm{c}}{\rm{i}}{\rm{r}}{\rm{c}}{\rm{u}}{\rm{m}}{\rm{f}}{\rm{e}}{\rm{r}}{\rm{e}}{\rm{n}}{\rm{c}}{\rm{e}}\,-\,(3.14\times {\rm{t}}{\rm{r}}{\rm{i}}{\rm{c}}{\rm{e}}{\rm{p}}{\rm{s}}\,{\rm{s}}{\rm{k}}{\rm{i}}{\rm{n}}{\rm{f}}{\rm{o}}{\rm{l}}{\rm{d}}\,{\rm{t}}{\rm{h}}{\rm{i}}{\rm{c}}{\rm{k}}{\rm{n}}{\rm{e}}{\rm{s}}{\rm{s}})$$

The measurement of triceps skinfold thickness was obtained using a Harpenden Skinfold Caliper. All measurements were taken on the right arm unless conditions were present that could interfere with the assessment (e.g., amputation, lymphedema, neurological diseases, recent upper extremity fracture, severe osteoarthritis with functional limitation).

Physical performance was assessed via the SPPB^25^, as previously described^39^. Briefly, the SPPB is composed of three timed subtests that evaluate standing balance, usual gait speed over a short track, and the ability to rise from a chair. For the standing balance test, participants were asked to stand in three increasingly challenging positions for 10 s each: standing with feet in side-by-side, semi-tandem, and full tandem positions. For the gait speed subtest, participants were asked to walk at their usual pace along a 4-m course, starting from a standing still position. The faster of two trials (m·s^−1^) was used for the calculation of the summary score. Finally, for the chair-stand subtest, participants were asked to rise from a chair and sit down five times as quickly as possible with arms folded across the chest. Each of the three SPPB subtasks was categorized into a five-level score according to predefined cut-points^25^, with 0 representing inability to do the test and 4 corresponding to the highest level of performance.

Upper extremity muscle strength was quantified using a hand-held hydraulic dynamometer (North Coast Medical, Morgan Hill, CA). As previously described^40^, participants performed one familiarization trial and one measurement trial with each hand, and the result from the stronger side was used for the analyses. Cognitive status was assessed using the six-item CPS^26^. CPS was scored on a 7-point ordinal scale, with higher scores indicative of worse cognitive performance.

Additional information about family history, lifestyle, physical activity and other behavioral factors was collected using questionnaires adopted in the *inCHIANTI* study^22^.

### Blood sample collection, genotyping and data processing

Fasting blood samples were obtained by venipuncture of the median cubital vein, using commercial collection tubes. Samples were processed for routine hematological and clinical chemistry tests. A whole-blood aliquot was frozen within 2 hrs of collection and kept at −80 °C until DNA extraction. Samples were thawed on ice and mixed thoroughly before use. Genomic DNA was extracted from peripheral blood according to standard procedures. Genotyping was performed in the extracted DNA for 561,490 SNPs on Infinium Human 610-QUAD version 1 (Illumina, CA, USA), according to the manufacturer’s protocol.

Analyses were limited to the 547,937 autosomal SNPs. We applied standard quality control procedures to the data, namely exclusion of samples with SNP call rates of less than 95%, samples with non-European ancestry, or samples demonstrating relatedness defined as identity-by-descent proportion of inheritance (pi_hat from the PLINK software toolset version 1.07 greater than 0.15)^41^, SNPs with minor allele frequency (MAF) less than 0.01, and SNPs with a Hardy-Weinberg equilibrium p value less than 0.001. The cryptic relatedness threshold led to the exclusion of individuals who shared more than 15% of their genome, which meant that related individuals down to third or fourth degree relatives were not included in the final analysis. The index individual whose sample had the best call rate from each related pair was selected for inclusion.

SNPs were then imputed to the June 2010 release of the 1,000 genomes project haplotypes using MaCH software (version 1.0)^42^. All SNPs passing quality control of MAF >0.05 and imputation quality from MaCH at >0.30 were included in the GWAS analyses. Genome-wide significance threshold was set at 5.0 × 10^−8^.

### Statistical analysis

Each genotyped and imputed SNP that passed quality control metrics was tested for genetic association with the following measurements using linear regression modeling: (1) ADL score (age, gender, number of diseases were included as covariates in the model); (2) MAMC (age, gender, number of diseases as covariates); (3) CC (age, gender, number of diseases as covariates); (4) SPPB summary score (age, gender, pain, number of diseases as covariates); (5) 4-meter walk test (age, gender, pain, number of diseases as covariates); (6) handgrip strength (age, gender, number of diseases as covariates); and (7) CPS (age, gender, number of diseases, depression, dementia as covariates).

In order to leverage the longitudinal nature of the *ilSIRENTE* dataset, we also analyzed changes over two years in (1) 4-meter walk test (age, gender, pain, number of diseases as covariates), (2) SPPB score (age, gender, pain, number of diseases as covariates), and (3) CPS (age, gender, number of diseases, depression, dementia as covariates). To avoid floor effect, individuals who scored 0 on the SPPB or who were unable to complete the 4-meter walk test at baseline were excluded. Each phenotype was transformed using quantile normalization to account for the non-normal distribution of some traits.

### Replication

The results of the genotyping analysis obtained from the *ilSIRENTE* cohort were compared with those of an existing genome-wide dataset generated for the *InCHIANTI* study^43^. The latter, similar to *ilSIRENTE*, is a study on the factors contributing to the decline in mobility and cognition in late life in two towns located in the Tuscany countryside (Italy)^22^. Genotype data were available for 184 persons over 80 years of age and 871 between 65 and 80 years.

### Data availability

The datasets generated during and/or analyzed during the current study are available from the corresponding author on reasonable request.
